# Supplementary material for: Novel Identification of the Collection of Pathogenic Fungal Species Verticillium with the Development of Species-Specific SSR Markers
Source: Pathogens. 2023 Mar 29;12(4):535. doi: 10.3390/pathogens12040535 (PMC10143602; doi:10.3390/pathogens12040535)
Supplement: Supplementary file 1 [file pathogens-12-00535-s001.zip › Supplementary/Table S1.pdf]

Supplementary Table S1: Designed and used LAMP primers for *V. nonalfalfae*/*V. dahliae* species differentiation

Name	LAMP region	Sequence	Species
Vnaa-Vd1_1	F3	GCGTCCAGAGACTACAGCT	*V. nonalfalfae*
	B3	AGCGGCAGATGAAATAGGTT	
	FIP	GATTCAAGGGCATGCACCGC-GCCAGATATCTGGGCAGC	
	BIP	TGCACATCAGGAGTTCAGGCAC-GCCAAGTTCTTTGGTGAGCT	
	F2	GCCAGATATCTGGGCAGC	
	F1c	GATTCAAGGGCATGCACCGC	
	B2	GCCAAGTTCTTTGGTGAGCT	
	B1c	TGCACATCAGGAGTTCAGGCAC	
	LoopF	CACTTGCAAAGCCTGTATTTCG	
	LoopB	GCTTTCCGGGTTATCCTTGGTT	
Vnaa-Vd1_2	F3	GCCGAAATACAGGCTTTGC	*V. nonalfalfae*
	B3	CCAACCAGCGATGTCACAAT	
	FIP	GTGCCTGAACTCCTGATGTGCA-GGTGCATGCCCTTGAATCC	
	BIP	ATTTCATCTGCCGCTCAGCGT-ACCCGACGTACGATAGAGG	
	F2	GGTGCATGCCCTTGAATCC	
	F1c	GTGCCTGAACTCCTGATGTGCA	
	B2	ACCCGACGTACGATAGAGG	
	B1c	ATTTCATCTGCCGCTCAGCGT	
Vnaa-Vd2	F3	TTCCTCCCATACGGGCAG	*V. nonalfalfae*
	B3	GGTGATACGCACCGAACAC	
	FIP	CTCCGCAGGCAAAGAAGCGT-GAATGAGCCATGTAGCCGAA	
	BIP	CGGTTGCGAAACACCAATCCC-GCGGTCAGGGTTTAATTCGA	
	F2	GAATGAGCCAtgTAGCCGAA	
	F1c	CTCCGCAGGCAAAGAAGCGT	
	B2	GCGGTCAGGGTTTAATTCGA	
	B1c	CGGTTGCGAAACACCAATCCC	
	LoopF	GTGCGCATCTACATGGATGG	
	LoopB	GAAGGAAGCAATCTCAGGATACTG	

Name	LAMP region	Sequence	Species
Vnaa-Vd3	F3	TCAGAGCGAGGGATGTGC	*V. nonalfalfae*
	B3	CCCTTTGCTGCAGTGGTATG	
	FIP	GTATGAAAGGGCTGCGAGGGG-CAGGTGATATCCGCAGTCAA	
	BIP	ATCAACTCCCCATGGGTGGC-TTCATCCACGGGGGCATT	
	F2	CAGGTGATATCCGCAGTCAA	
	F1c	GTATGAAAGGGCTGCGAGGGG	
	B2	TTCATCCACGGGGGCATT	
	B1c	ATCAACTCCCCATGGGTGGC	
	LoopF	GCATTCCAATCTCCTacCTAACAC	
	LoopB	CTCTGCAGTGAAACTCCAATCA	
PG2_1	F3	CGAATCACGGAGACTGAGAC	letalna oblika *V. nonalfalfae*
	B3	ACTTTAATCGCCCCGTCTG	
	FIP	CATGGGGCCATGGAAGCACG-TGGATGTCGGGCAGAGTT	
	BIP	GCGCAGGCTAGGCGTTCTTT-CAGCCAGTATGCGAACCAAT	
	F2	TGGATGTCGGGCAGAGTT	
	F1c	CATGGGGCCATGGAAGCACG	
	B2	CAGCCAGTATGCGAACCAAT	
	B1c	GCGCAGGCTAGGCGTTCTTT	
	LoopF	GACTAAGCCGTCGCAGGT	
	LoopB	CACTCGCGAGAAGCCAGGA	
PG2_2	F3	TCGAGCGCTTTCTATCCTCA	letalna oblika *V. nonalfalfae*
	B3	TGTTGAACGGATGCAGATCG	
	FIP	TGGTCCCATCGGGGCAATACAT-CTCATCCACAACAGGGGC	
	BIP	TATGTCCGAGAGATGCAGCAGC-GATGACGACGAGCAACCTC	
	F2	CTCATCCACAACAGGGGC	
	F1c	TGGTCCCATCGGGGCAATACAT	
	B2	GATGACGACGAGCAACCTC	
	B1c	TATGTCCGAGAGATGCAGCAGC	
	LoopF	GGTCAATGGTTGGGTATCACC	
	LoopB	GAGTGATTGGTGGATGGAAATGC	
Vd-mitoh.	F3	ACTCTTCGAATTCTCTCTTCT	*V. dahliae*
	B3	GTTTAAGTGAGCATACTGTCAT	
	FIP	TAGAGAGATTCGACGTTACGAGAAT-TATTTGGGTAAGTATAGTAATGAGG	
	BIP	TACTCTTGTCCCTTAAAATTTCGCT-AGTTACTACCTTAAATAAGGAAACG	
	F2	TATTTGGGTAAGTATAGTAATGAGG	
	F1c	TAGAGAGATTCGACGTTACGAGAAT	
	B2	AGTTACTACCTTAAATAAGGAAACG	
	B1c	TACTCTTGTCCCTTAAAATTTCGCT	

Name	LAMP region	Sequence	Species
Vnaa-mitoh.	F3	GTATCTGTATAGAATTCTACACTCA	*V. nonalfalfae*
	B3	GAGTTAATGTAACATTCTTCCCA	
	FIP	AGTGTTGTAGCTGCTTGATTATTCT-TTCAAGGATTTCTGCTTGCA	
	BIP	GTTTCAACCTGCGTAAGCGT-GGATTACAAGGAATGCCTAGA	
	F2	TTCAAGGATTTCTGCTTGCA	
	F1c	AGTGTTGTAGCTGCTTGATTATTCT	
	B2	GGATTACAAGGAATGCCTAGA	
	B1c	GTTTCAACCTGCGTAAGCGT	
12_1_Chr2	F3	CGTCCAAATGACTGTGCTCA	*V. nonalfalfae*
	B3	AGGGTTGATGGGGAAGGAG	
	FIP	CGGCGCTCATCGACAGTCTC-GACACATCTCTCGGGACCA	
	BIP	TGACCTATTGCCCCGAAACTCC-CCCCATATCCGACATGATCC	
	F2	GACACATCTCTCGGGACCA	
	F1c	CGGCGCTCATCGACAGTCTC	
	B2	CCCCATATCCGACATGATCC	
	B1c	TGACCTATTGCCCCGAAACTCC	
	LoopF	GCTGTGCTTGACCAGGAGC	
	LoopB	TTCAAAAGCCAAGGAACTACATGG	
14Chr3	F3	TCCCGTCGCAAGATACCG	*V. nonalfalfae*
	B3	ACTCGAGTTCGTAGCTCAGG	
	FIP	TTCCGACCTCTCAGCGCTCG-GCGCCCTTCCATTCCCTA	
	BIP	GAGACCGACGCGGCAAAGAT-CCCTCAATGACAATGTCCCG	
	F2	GCGCCCTTCCATTCCCTA	
	F1c	TTCCGACCTCTCAGCGCTCG	
	B2	CCCTCAATGACAATGTCCCG	
	B1c	GAGACCGACGCGGCAAAGAT	
	LoopF	GCTTTGAGCTGTGGCTTCC	
	LoopB	TTCAAAAGCCAAGGAACTACATGG	
