# Supplementary material for: Novel Identification of the Collection of Pathogenic Fungal Species Verticillium with the Development of Species-Specific SSR Markers
Source: Pathogens. 2023 Mar 29;12(4):535. doi: 10.3390/pathogens12040535 (PMC10143602; doi:10.3390/pathogens12040535)
Supplement: Supplementary file 1 [file pathogens-12-00535-s001.zip › Supplementary/Table S3.pdf]

Supplementary Table S3: Diversity by species

**Species**		**Na**	**N**	**Ne**	**I**	**h**	**uh**
V. nonalfalfae		29					
	Mean	2.90	49.70	1.11	0.22	0.09	0.09
	SE	0.31	0.21	0.03	0.04	0.02	0.02
V. alfalfae		21					
	Mean	2.10	5.00	2.01	0.71	0.50	0.62
	SE	0.10	0.00	0.09	0.04	0.02	0.02
V. dahliae		27					
	Mean	2.70	19.90	1.41	0.43	0.22	0.23
	SE	0.42	0.62	0.17	0.12	0.06	0.07

Na = No. of different alleles

N = Size of population

Ne = No. of effective alleles = 1 / (Sum pi^2)

I = Shannon's Information Index = -1* Sum (pi * Ln (pi))

h = Diversity = 1 - Sum pi^2

uh = Unbiased Diversity = (N / (N-1)) * h

(Where pi is the frequency of the ith allele for the population & Sum pi^2 is the sum of the squared population allele frequencies.)
