# Supplementary figures and images for: Novel Identification of the Collection of Pathogenic Fungal Species Verticillium with the Development of Species-Specific SSR Markers
Source: Pathogens. 2023 Mar 29;12(4):535. doi: 10.3390/pathogens12040535 (PMC10143602; doi:10.3390/pathogens12040535)

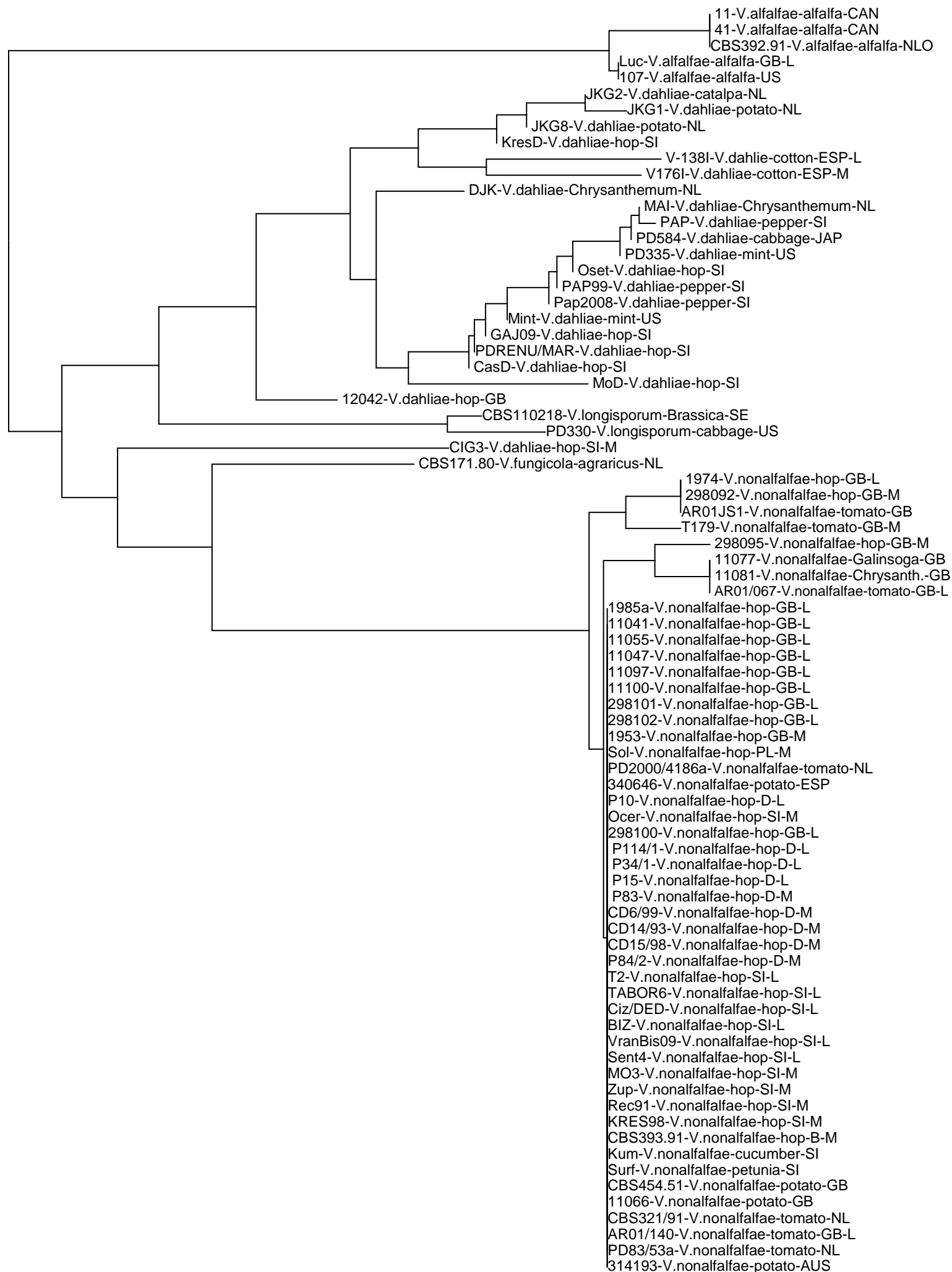

**Figure S1:** SSR marker analysis based phylogenetic tree

Supplement: Supplementary file 1 [file pathogens-12-00535-s001.zip › Supplementary/Figure S1.pdf]
